# Using Latent Class Analysis to Explore Complex Associations Between Socioeconomic Status and Adolescent Health and Well-Being

**DOI:** 10.1016/j.jadohealth.2021.06.013

**Published:** 2021-11

**Authors:** Emily Lowthian, Nicholas Page, G.J. Melendez-Torres, Simon Murphy, Gillian Hewitt, Graham Moore

**Affiliations:** a Population Data Science, Swansea University Medical School, Swansea, UK; b DECIPHer, School of Social Sciences, Cardiff University, Cardiff, UK; c South Cloisters, University of Exeter Medical School, Exeter, UK; d Wolfson Centre for Young People’s Mental Health, Cardiff University, Cardiff, UK

**Keywords:** Latent class analysis, Socioeconomic status, Mental health, Substance use

## Abstract

**Purpose:**

Research demonstrates a strong socioeconomic gradient in health and well-being. However, many studies rely on unidimensional measures of socioeconomic status (SES) (e.g. educational qualifications, household income), and there is often a more limited consideration of how facets of SES combine to impact well-being. This paper develops a multidimensional measure of SES, drawing on family and school-level factors, to provide more nuanced understandings of socioeconomic patterns in adolescent substance use and mental well-being.

**Methods:**

Data from the Student Health and Wellbeing Survey from Wales, UK was employed. The sample compromised 22,372 students and we used latent class analysis to identify distinct groups using three measures of SES. These classes were then used to estimate mental well-being, internalizing symptoms, and substance use.

**Results:**

The five-class solution offered the best fit. Findings indicated distinct classes of families as follows: “nonworking,” “deprived working families,” “affluent families in deprived schools,” “lower affluence,” and “higher affluence.” There was a clear relationship among the classes and mental well-being, internalizing symptoms, smoking, and cannabis use; alcohol was the exception to this.

**Conclusions:**

The identification of these classes led to a fuller understanding of the health and well-being effects of SES, showing clearer patterning in health behaviors that often is not captured in research. The implications for adolescent health and well-being are discussed, including considerations for future research.

**Implications and Contribution**

Through latent class analysis, this study identified five distinct classes of underlying SES patterns and dynamics. These classes predicted mental well-being and substance use, and two classes (10% of sample) were at near double the risk for greater substance use, internalizing symptoms, and poorer mental well-being.

See Related Editorial on p.685

Longitudinal and cross-sectional research shows a socioeconomic gradient throughout the life course in health and well-being outcomes worldwide [[1], [2], [3], [4]]. More specifically, improved mental health and well-being is often observed in groups with higher socioeconomic status (SES). Similarly, links between SES and substance use are well evidenced in a substantial body of literature. However, associations vary depending on both the substance (e.g., alcohol, tobacco, or cannabis) and its measurement (e.g., initiation or more regular use). Indeed, while people in lower SES groups are consistently shown to be more likely to smoke tobacco, associations with alcohol use tend to be more inconsistent, with some epidemiological evidence observing a higher likelihood among more affluent groups [3,5,6]. Such inconsistencies have likewise been demonstrated for associations between SES and illicit drug use [3], with some studies suggesting that higher socioeconomic groups are more likely to use illicit substances [[6], [7], [8]] and vice-versa [9]. The onset of these associations are as early as childhood and adolescence [10,11], and given that adolescence is a critical window in the emergence of mental health issues, and the development of health behaviors which are carried into adulthood, this must be fully understood [12].

However, defining SES is a challenge in research as it is multifaceted. For instance, educational qualifications are knowledge assets, whereas income is a material asset, which promotes safe housing, healthy food, and exercise [13]. Although all SES measures capture an uneven distribution of power and status, the effect sizes that follow can be an artifact of the type of measurement used (e.g., employment, neighborhood disadvantage). This variation in measurement has affected wider interpretations of the impact of SES on young people’s health and well-being [6,10]. For example, household income has been positively associated with adolescent alcohol use, while maternal education was inversely associated [14]. Here it was suggested that the use of family income as a measure of SES might capture the availability of (and hence access to) alcohol via financial means [14]. Therefore, the exclusive use of family SES ignores the impacts of school or neighborhood SES. Cross-sectional analysis of a national survey in Wales shows that school-level SES, such as free school meal entitlement (FSM), has independent impacts on adolescent well-being [[15], [16], [17]]. Moreover, young people from lower SES households have less access to structural resources to prevent symptoms from risk behaviors [10].

Given the mixed literature, a measure that identifies patterns of SES from multiple assets and structures should be developed. This will enable an understanding of SES dynamics, specifically how measures of SES relate to one another, and develop groups that represent underlying socioeconomic circumstances. These groupings can then be used to predict areas of child health and well-being. An example of this work is Skogen et al [18]. They employed latent class analysis using various longitudinal measures, including both parental employment status and educational attainment. Here the authors identified four distinct SES groups: “never poor,” “chronically poor,” and two groups which fluctuated in and out of poverty. The chronically poor group reported less daily cigarette smoking (or snus use), less alcohol use, and were less prone to a substance use related ill health. In contrast, the groups which moved in and out of poverty were more likely to use illicit drugs and drink alcohol compared to those classed as “never poor,” who themselves were less likely to have used illicit drugs or to smoke daily. Constructs other than SES have also been developed using latent class analysis to understand young people’s behavior. For instance, Valente et al. [19] used latent class analysis to derive substance use, and found that authoritative, authoritarian, and indulgent styles of parenting predicted the adolescent poly-use class, as did high SES. Furthermore, positive health behaviors, specifically physical activity and fruit and vegetable consumption were somewhat socioeconomically patterned, with education status being a key predictor in latent class models [20].

As the study by Skogen et al. developed a greater understanding of SES patterns in health behaviors, this study aims to take advantage of one of the largest population samples of secondary school-aged children in Europe to better understand how family and school-based SES measures predict adolescent substance use and mental well-being. Specifically, through means of latent class analysis, we examine (1) whether family and school-level measures could be integrated to form a more holistic measure of SES, and (2) subsequent associations between latent classes and adolescent well-being and substance use outcomes.

## Methods

### Student and school-level data

Adolescent self-reported data were obtained for secondary analysis from the 2017 Student Health and Wellbeing (SHW) survey [21], completed by 11–16 year olds in Wales by the School Health Research Network at Cardiff University [21]. It is an electronic, closed response, self-completion survey encompassing a wide range of questions on adolescent health and other risk-related behaviors. As of 2017, the World Health Organisation’s Health Behaviour in School-aged Children Wales Survey is also embedded within the SHW survey. In 2017, all maintained secondary schools in Wales were members of the School Health Research Network and were invited to take part in the survey. In 2017, 193 schools participated in data collection, representing 91% of eligible secondary schools. Consent was required at three levels: school, parent, and student. Questions on parental employment were only asked to students attending Health Behaviour in School-aged Children–designated schools in 2017. As a result, the analytical sample used in this study is reduced, representing around a quarter of responses. School-level data from the Pupil Level Annual School Census was accessed online (see www.statswales.gov.wales) and linked to sampled schools to attain information on school-level deprivation.

### Ethics

Permission to access the data was granted by the data owners of the SHW survey. Informed consent was obtained from schools, parents, and students. Schools had to be registered to take part in the survey, parents could withdraw their child(ren), and the student’s participation was optional, with the first question in the survey asking for their consent.

### Measures

#### Socioeconomic status

##### The family affluence scale

The Family Affluence Scale (FAS) is a composite measure of affluence based on six questions exploring car, computer, and dishwasher ownership, bedroom occupancy, frequency of family holidays, and the number of household bathrooms. Commonly used in social research, FAS is considered to be a robust tool for identifying socioeconomic differences within adolescent cohorts [22,23].

##### Parental employment status

Students were asked about their mother’s and father’s employment status. Responses included the following: mother is employed, not employed but looking for a job, and they are retired, a carer, or has long-term health conditions; these questions were repeated for fathers. Children who said they did not have, or saw, their mother or father were set to missing.

##### Free school meal entitlement

School-level SES was measured using the average FSM entitlement of the school: a statutory benefit available to students whose families receive some form of income-related benefits (e.g., Jobseeker's Allowance) [24]. Here, a greater proportion of students eligible to receive FSM is indicative of lower SES school intakes and vice versa.

#### Covariates

##### Gender

The responses included “Boy,” “Girl,” and “I do not want to answer”; gender was analyzed as male or female students, with “I do not want to answer” set to missing.

##### School year

This constitutes which year group students were in (year 7–11) when students are aged between 11 and 16 years old.

##### Ethnicity

Seven groups of ethnicity were used: White, White traveler, Mixed ethnicity, Indian or Bangladeshi or Pakistani, Chinese, Black African or Caribbean, Arab, and Other.

#### Outcome measures

##### Student mental health

Student mental well-being was captured using two measures: (1) the Short Warwick-Edinburgh Mental Well-Being Scale and (2) a composite measure of internalizing behavior. The Short Warwick-Edinburgh Mental Well-Being Scale is a widely used and validated well-being measure (which has recently been validated within the study sample) [25,26]. It is derived from responses to seven positively worded questions (e.g., “I've been feeling relaxed,” “I've been feeling close to other people”) and asks students the extent to which they have felt this way over the past 2 weeks (response options: “none of the time,” “rarely,” “some of the time,” “often,” “all of the time”). Responses are summed to create an overall mental well-being score, where higher scores reflect greater well-being. As an indicator of internalizing behavior, students were asked the extent to which they have felt low, nervous, irritable, or bad tempered, or experienced difficulty sleeping over the past 6 months (response options: “about every day,” “more than once a week,” “about every week,” “about every month,” “rarely or never”). Again, responses were summed to create an overall scale score, with higher scores reflecting poorer outcomes.

##### Substance use

Students were also asked at what age they first smoked a cigarette (more than a puff), used cannabis, and drank alcohol (more than a small amount). For each substance, a binary measure indicating ever use was defined whereby any evidence of prior use (i.e., age at initiation between 11 and 16 years) was coded “yes.” Drawing on questions of substance use frequency, regular smoking was classed as daily or weekly use, while recent use of cannabis was classed as any use in the past 30 days, and for alcohol, the frequency of drunkenness in the past month was used [27].

### Data analysis

Data were managed, and descriptive statistics were analyzed using Stata 15.2 [28]; the analysis was conducted in Mplus version 8.4 [29]. Our analysis strategy unfolded in several steps. First, factor analysis of the FAS measures was conducted to explore factor structure. Second, the latent class analysis was fitted with the FAS items as a latent variable and the manifest variables of mother’s and father’s employment and school-level FSM percentage (%). Third, the classes were then predicted by covariates. Finally, the latent classes were used to predict distal outcomes.

### Factor analysis

FAS items were used to form a latent variable, which is advantageous over a sum-score method as it estimates measurement error [30]. Using a polychoric correlation matrix, we first estimated a principal components model to verify the number of factors. We then estimated an Exploratory Factor Analysis model using a weighted least squares mean and variance estimator, as recommended for categorical data [30]. Model fit estimates included the root mean square error of approximation, the comparative fit index, the Tucker-Lewis fit index, and standardized root mean squared residual. We also inspected factor loadings (above ±.40) [30,31].

### Latent class analysis

Latent class analysis was used to explore unobserved constructs in observed data [25]. This technique uses multiple variables to identify the presence of underlying classes, or groups in the data. A latent class model was performed first to understand the number of classes that generated an optimal solution for the data. We then related these latent classes to distal outcomes.

To estimate the optimal number of classes, we used maximum likelihood parameter estimates with standard errors approximated by first-order derivatives and a conventional chi-square statistic, recommended by Muthén and Muthén [29] (Table S3). Models with two, three, four, five, and six latent classes were estimated. Assessment for model fit was undertaken by balancing interpretability and fit criteria [25]. Three statistical criteria were used to assess model fit [31]: Bayesian information criterion; scaled relative entropy, a measure whereby “0% indicates very poor certainty in classification, and 100% indicates perfect certainty” [25]; and the Bootstrap Likelihood Ratio Difference Test [29]. To estimate the effect of covariates, the method of Bolck, Croon, and Hagenaars was used as recommended [29,32]. For distal outcomes, the automatic three-step method [29,33,34] was used to assign observations to their most likely class, estimate misclassification matrices, and then use these estimates to calculate equality of means and probabilities across the classes.

## Results

### Study sample

The analytical SHW survey sample consisted of 22,372 students from 83 secondary schools (88 were initially invited in 2017), who were a subsample of the overall survey who were asked questions on parental employment (Table 1). Overall, the sample was evenly distributed by gender (female: 51%) and less even across school years, with Year 9 being the most common (23%), and Year 11 the least (17%). Most students were of White ethnicity (91%). Student sociodemographic characteristics differed little between the subsample included in this analysis and the full SHW sample (Supplementary Table S1).

**Table 1:** Sample demographics and health outcomes

	N/mean/median (%/SD)
Gender (n = 22,372%, 100%)	
Male	10,710 (48%)
Female	11,314 (51%)
I do not want to answer	348 (2%)
School year (n = 22,372%, 100%)	
Year 7	4,340 (19%)
Year 8	4,762 (21%)
Year 9	5,060 (23%)
Year 10	4,391 (20%)
Year 11	3,819 (17%)
Ethnicity (n = 21,749%, 97%)	
White	19,746 (91%)
White, traveler	138 (1%)
Mixed ethnicity	502 (2%)
Indian/Bangladeshi/Pakistani	410 (2%)
Chinese	70 (<1%)
Black African or Caribbean	245 (1%)
Arab	123 (1%)
Other	515 (2%)
Ever smoking (n = 22,073%, 99%)	
Yes	2,789 (13%)
Ever alcohol (n = 21,965%, 98%)	
Yes	9,268 (43%)
Ever cannabis (n = 22,023%, 98%)	
Yes	1,780 (8%)
Regular smoking (n = 21,604%, 97%)	
Yes	683 (3%)
Regular alcohol (n = 21,935%, 98%)	
Yes	2,067 (9%)
Past-month cannabis (n = 21,926%, 98%)	
Yes	965 (4%)
WEMWBS (n = 22,372%, 100%)	21.90/21.54 (4.52)
Internalizing (n = 22,372%, 100%)	5.66/5.00 (4.47)

WEMWBS = Warwick-Edinburgh Mental Well-Being Scale.

Regarding SES measures, most students reported family access to a car (95%), going on holiday at least once or more per year (87%), having their own bedroom (85%), and possessing more than two computers (75%), while almost two thirds stated their family had a dishwasher (62%). The majority of students also reported having up to two household bathrooms (81%). Most mothers were employed (85%), although father employment was higher (94%). A small percentage of students reported their parents as currently seeking employment, with a greater proportion of mothers (3%) than fathers (1%) in this category. Likewise, mothers were more likely to be carers or retired (12%) compared to fathers (5%). At the school level, mean FSM entitlement was 17%.

### Factor analysis

Principal components analysis suggested that a one-factor model was best, estimating an eigenvalue of 1.89; subsequent factors had eigenvalues <1.00. Component values ranged between .29 and .48, with bedrooms being the lowest and bathroom being the highest. The model fit for the Exploratory Factor Analysis was good (root mean square error of approximation = .03, 90% confidence interval = .03–.04, comparative fit index = .98, Tucker-Lewis fit index = .97, standardized root mean squared residual = .03). The factor loadings were adequate, and average standardized factor loadings were .51, ranging from .38 to .64. Having a dishwasher, the number of bathrooms, and number of cars loaded higher (.64 and .59) compared to the number of computers, having their own bedroom, or the number of holidays (.38, .41, and .42 respectively).

### Latent class analysis

The five-class solution showed the best model fit overall. Although the six-class model had the lowest Bayesian information criterion value, the model could not replicate the best log-likelihood up to 400 random starts (all other models used 100), therefore the model was rejected. The entropy increased as the classes increased, with the five-class solution’s entropy being the highest (.74) for all successful models. Only the four- and five-class entropy was acceptable, >.70 as recommended [35]. For classification accuracy, models should be higher than .80 (or 80%) for each class [36], all models showed classes with less than this but did not warrant concern for model reliability. Furthermore, the Bootstrap Likelihood Ratio Difference Test suggested that five classes were better than four. Therefore, the five-class solution was considered as the best solution, balancing statistical criteria and theoretical interpretation [37].

### Sample proportions and means of five-class solution

#### Class 1: nonworking families (3%)

This class compromised children from families where nearly all mothers and fathers were retired, had a caring responsibility, or a long-term health condition (99% and 98%, respectively). The average FSM of schools attended by students within this group was similar to the population average (16%, standard deviation [SD] 5.80), suggesting that FSM was not a key predictor of this class. These children were from families with slightly lower than average affluence, with almost half of children reporting that their family had two or more cars (49%), having their own bedroom (80%), having more than two computers (67%), near half having a dishwasher (45%), two thirds had one bathroom (65%), and most went on holiday at least once or more (83%).

#### Class 2: affluent families in deprived schools (13%)

This class compromised children who, on average, attended schools with higher FSM intake than the population average (30%, SD 4.63), but were from families with typical or slightly above average affluence. Most children had access to two or more cars (71%), had their own bedroom (89%), more than two computers (77%), a dishwasher (68%), while a small majority reported having two or more bathrooms (56%) and reported going on holiday at least once or more (90%). Most mothers and fathers were employed (91% and 99%, respectively), with a small number looking for a job (2% and 1%) and 7% of mothers were retired or carers.

#### Class 3: lower affluence families (35%)

The average FSM of schools attended by students within this group was similar to the overall population average (15%, SD 5.19). However, children within this class were from families with lower than average affluence, with approximately half of children reporting that their family owned one car (49%), most had their own bedroom (78%), had more than two computers (65%), less had a dishwasher (39%), most had only one bathroom (70%), and most went on holiday at least once or more (81%). Most mothers and fathers were employed (86% and 97%, respectively), with a moderate percentage looking for a job (5% and 2%) and 10% of mothers were retired or carers (compared to 1% of fathers).

#### Class 4: higher affluence families (41%)

This class compromised children who, on average, attended schools with the lowest FSM average (12%, SD 4.81). These children had the highest family affluence scores, with most children having access to two or more cars (89%), most had their own bedroom (95%), had more than two computers (86%), had a dishwasher (88%), most had two or more bathrooms (80%), and only 6% did not go on holiday that year, whereas 55% went more than twice. Most mothers and fathers were employed (95% and 99%, respectively), with a small percentage looking for a job (1% and 0%, respectively) and 5% of mothers were retired or carers (compared to 1% of fathers).

#### Class 5: deprived families (7%)

This class compromised children who attended schools with the highest FSM average (34%, SD 6.47). These children had the lowest affluence scores, with 15% having access to no car, the highest proportion of children not having their own bedroom (34%), the lowest proportion of having more than two computers (56%), only 24% had a dishwasher, the most common answer was one bathroom (81%), and 26% did not go on holiday that year. Just over half of mothers were employed (56%) compared to near four fifths of fathers (78%). Just over a third of mothers were retired or carers (36%), this was halved for fathers (18%). This class had the highest proportion of mothers and fathers looking for a job (8% and 5%, respectively).

For class proportions and comparison to sample averages, see Supplementary Table S4.

### Demographics

The higher affluence class was used as the reference class (Table 2). Females were more likely to be in the deprived (odds ratio [OR] 1.24, *p* < .05) and nonworking class (OR 1.23, *p* < .05). Older students were more likely to be in the lower affluence class (OR 1.15, *p* < .05), deprived (OR 1.09, *p* < .05), and nonworking class (OR 1.13, *p* < .05). Those who did not identify as White were more likely to be in the affluent families in deprived schools class (OR 1.15, *p* < .05), lower affluence class (OR 1.18, *p* < .05), and deprived class (OR 1.23, *p* < .05), but not the nonworking class.

### Well-being and substance use

**Figure 1:**
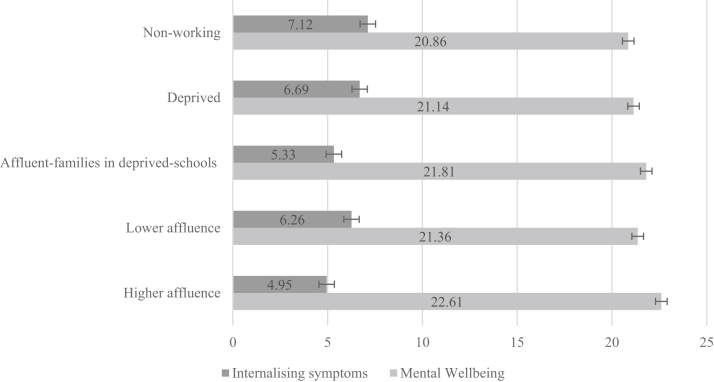
Mental well-being and internalizing symptoms means and standard error bars for each class.

Mental well-being was lowest among the nonworking families and was highest in higher affluence families (Figure 1). The deprived, lower affluence, and affluent families in deprived schools classes were similar. Likewise, the nonworking class had the highest mean for internalizing symptoms; the socioeconomic patterning for this variable mirrored the mental well-being outcome.

**Figure 2:**
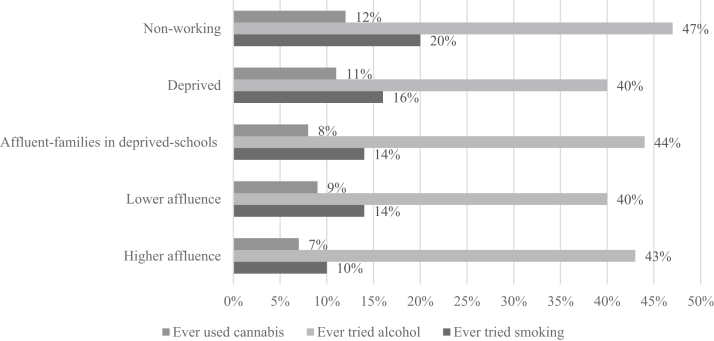
Proportions of classes who have used tobacco, alcohol, or cannabis.

For ever use of substances, the class identified as “nonworking” had the highest proportion of ever trying smoking and cannabis, and the deprived class (Figure 2) followed this. The higher affluence class had the lowest proportions, followed by the lower affluence class and affluent families in deprived schools class. Patterning in alcohol use was less clear, but the nonworking class had the highest proportion, followed by the affluent families in deprived schools class and then the higher affluence class. The deprived and lower affluence classes had the lowest proportions.

**Figure 3:**
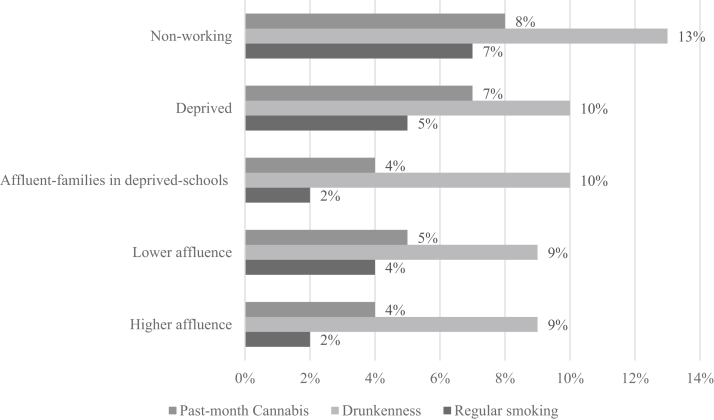
Proportions of classes who use tobacco regularly, or have used cannabis or been drunk in the last 30 days.

For regular substance use, the nonworking class had the highest proportions of smoking, drunkenness, and past-month cannabis use; therefore, the differences in drunkenness were small (Figure 3). For smoking, there were clear differences, as the higher affluence and affluent families in the deprived schools class had the lowest proportion, and the deprived and lower affluence classes had double the proportion. For cannabis, the patterning was the same, with the higher affluence and affluent families in deprived schools class being the lowest, and the deprived and lower affluence classes being higher. Near all equality of the mean, and Wald tests were statically significant, aside from drunkenness (Supplementary Table S6).

## Discussion

We show that simultaneously using multiple socioeconomic indicators across varying socioecological levels can reveal more nuanced understandings of the association of SES with adolescent health and well-being. This differs from intercorrelation-based understandings, that is, multilevel modeling and enables a different understanding of SES dynamics and patterning. Likewise, the FAS was confirmed as unidimensional, and fit for use as a latent variable. Drawing on measures of family affluence, parental employment, and school-level deprivation collected from a large national sample of adolescents in Wales, UK, we identified five distinct latent classes of SES: “nonworking families,” “affluent families in deprived schools,” “lower affluence families,” “higher affluence families,” and “deprived families” and explored how these classes differed according to demographics and predicted well-being and substance use outcomes.

Our findings mirror much of the research on the impacts of SES on mental well-being [10], and substance use [9]. However, this approach enabled two novel dimensions of SES that are less captured by research using single or multiple SES measures—the nonworking families class and affluent families in deprived schools class. First, the nonworking families are likely to be those at the more extreme ends of poverty, similar to the “chronically poor” class identified in Skogen et al. [18]; these are households where parents cannot work and may include circumstances where children need to care for their parents due to illness, and this perhaps captures elements of social isolation. Most concerningly, these children had the poorest health indicators across *every* outcome. This supports other research using this survey that average mental well-being was positively associated with family affluence. However, that report evidenced higher mental well-being scores for each tertile of family affluence compared to our study, for example, nonworking class (20.9) versus the least affluent tertile (22.0) [38]. In terms of substance use, the gradient found in smoking was supported [38] and we confirmed lower SES classes had a higher proportion of cannabis use in the past month. However, we also found that drunkenness was higher among lower SES classes too. This contrasts with research which suggests that alcohol is used by higher SES groups [6,14,16], which alludes to the problems associated with SES measurement. Therefore, modeling relationships using a single SES measure may obscure impacts on those at the extreme ends of the socioeconomic spectrum, who are most at need.

Second, the affluent families in deprived schools class encompassed students with high family affluence who attended deprived schools. These students had average mental well-being (21.81 compared to sample average of 21.90) and internalizing symptoms (5.33 vs. 5.66), but the initiation of alcohol and drunkenness was the second highest and mirrored the deprived class. This may elucidate the family-school SES complexity whereby these students possess cultural behaviors more aligned with their deprived peers in school, but have more access to “socially acceptable” substances such as alcohol, as seen in other studies [6,14,16]. For instance, a study using the 2010 survey in Wales found that family affluence had a positive association with alcohol (OR 1.08, *p* < .05), but school affluence was negative (OR .87, *p* < .05) [16]. Outside of this, other family SES measures, such as parental education, are argued to be inversely related to alcohol use, confirming the influence parents have on their children [39]. Hence, our findings support Moore and Littlecott [16], emphasizing that school and family SES exerts independent and combined influences upon adolescent health behaviors. However, the technique used can only create classes and is unable to test specific dimensions of SES, unlike approaches that test multiple SES measures in a single model.

The other three classes were more in line with what is currently measured as SES—lower affluence, higher affluence, and deprived families. Although the higher affluence families had the most positive behaviors, they did use alcohol in similar amounts to other classes; this mirrors other literature which suggests that affluence is related to access [6,14]. However, we found that drunkenness was the lowest in this class. The lower affluence families and deprived classes had outcomes consistent with the literature, particularly in terms of smoking [6,14] and cannabis [6]. For smoking, the proportion of regular smokers was slightly lower in the deprived group (5%) compared to lowest FAS tertile (6%) shown in Hewitt et al. [38]; the lower affluence group mirrored the mid FAS tertile (4%). For cannabis, ever use was higher in the deprived groups than report estimates for the lowest tertile (11% vs. 9%), whereas the lower affluence mirrored the report again (9%) [38]. These findings suggest that using FAS tertiles alone may obscure SES gradients in well-being, and not fully highlight those in need.

Although our research illuminates the importance of measuring SES at multiple socioecological levels, it would benefit from longitudinal measures as in Skogen et al., as a cross-sectional study can only give associations. Linkage to more objective data on parental employment, education level, and student-level FSM would be beneficial, along with measures of neighborhood deprivation, despite that research suggests that neighborhood deprivation does not operate in same manner as individual measures, and cannot be combined in a linear fashion [40]. Much of our study draws on self-reported behaviors which are known to introduce different biases; we attribute the ORs found in Table 2 to females being less likely to introduce self-report bias, and older students being more informed about their parent’s circumstances. In addition, we acknowledge the temporal complexity with using age initiation measures to describe “ever” alcohol, cannabis, or tobacco use, as we cannot confirm that SES is stable over-time and warn readers to interpret these findings as associations rather than causal relationships. Moreover, our study uses more traditional measures of SES for family employment, which does not best represent those in kinship or foster care. We also acknowledge that measurement invariance of FAS and the latent class analysis should be considered for model generalizability. Going forward, we note the age variability with substance use, and future research should explore age-specific outcomes, and other areas of well-being such as diet, exercise, and social support.

**Table 2:** Odd ratios of being in a class

	Higher affluence (41%)	Affluent families in deprived schools (13%)	Lower affluence (35%)	Deprived (7%)	Nonworking (3%)
Gender	Reference	.92	1.00	**1.24**	**1.23**
School year	Reference	1.02	**1.15**	**1.09**	**1.13**
Ethnicity	Reference	**1.15**	**1.18**	**1.23**	1.06

Bold values represent *p* < .05.

Although our study has some shortcomings, we conclude that multidimensional measures have further informed our understanding of SES and its impact on adolescent mental well-being and substance use, specifically in terms of alcohol where the literature is less clear. The use of latent class analysis has revealed a socioeconomic gradient in adolescent health and well-being, and although it is not clear how that operates, this evidences the need for better developed SES measures in research. Our findings suggest that 3% of all secondary school students in Wales are twice as likely to be at risk for poor health and well-being (the nonworking class), and 7% are at a higher risk (the deprived class). Therefore, 10% of secondary school students in Wales within these two identifiable SES classes, have near double the risk for poorer health and well-being outcomes.
