# Supplementary material for: Using Latent Class Analysis to Explore Complex Associations Between Socioeconomic Status and Adolescent Health and Well-Being
Source: J Adolesc Health. 2021 Nov;69(5):774–81. doi: 10.1016/j.jadohealth.2021.06.013 (PMC9225957; doi:10.1016/j.jadohealth.2021.06.013)
Supplement: Supplementary Tables [file mmc1.docx]

**Supplementary materials**

| **2017 HBSC/SHW full survey sample demographics** | |
| --- | --- |
|  | **N (%)** |
| **Gender** *(n=103,971, 100%)* | |
| Male | 50,452 (48.5%) |
| Female | 51,458 (49.5%) |
| Prefer not to say | 2,061 (2.0%) |
| **School year** *(n=103,971, 100%)* | |
| Year 7 | 22,634 (21.8%) |
| Year 8 | 22,421 (21.6%) |
| Year 9 | 22,208 (21.4%) |
| Year 10 | 19,704 (19.0%) |
| Year 11 | 17,004 (16.4%) |
| **Ethnicity** *(n=100,448, 97%)* | |
| White | 86,000 (85.6%) |
| White – traveller | 877 (0.9%) |
| Black and minority ethnic | 13,571 (13.5%) |

*Table S1: Full sample demographics and outcomes*

*Table S2: Reduced sample socioeconomic status measures*

|  | **N** | **Mean/%** | **% Missing** |
| --- | --- | --- | --- |
| **FSM** | 22,372 | 17% | 0% |
| **Family Affluence Scale (imputed scale)** | | | |
| *Do you have access to a car?* | | | |
| No | 1035 | 5% | 0% |
| Yes, one | 6821 | 30% |  |
| Yes, two or more | 14516 | 65% |  |
| *Do you have your own bedroom?* | | | |
| No | 3175 | 14% | 0% |
| Yes | 19197 | 85% |  |
| *How many computers do you have at home?* | | | |
| None | 403 | 2% | 0% |
| One | 1805 | 8% |  |
| Two | 3487 | 16% |  |
| More than Two | 16677 | 75% |  |
| *Do you have a dishwasher at home?* | | | |
| No | 8460 | 38% | 0% |
| Yes | 13912 | 62% |  |
| *How many bathrooms do you have at home?* | | | |
| None | 53 | <1% | 0% |
| One | 10447 | 47% |  |
| Two | 7704 | 34% |  |
| More than two | 4168 | 19% |  |
| *How many holidays have you been on this year?* | | | |
| Not at all | 2969 | 13% | 0% |
| Once | 5465 | 24% |  |
| Twice | 5052 | 23% |  |
| More than twice | 8886 | 40% |  |
| **Does your mother have a job?** | | | |
| Yes | 19047 | 85% | 2725 (12%) |
| No – looking for a job | 600 | 3% |  |
| No – retired or is a carer | 2725 | 12% |  |
| **Does your father have a job?** | | | |
| Yes | 18594 | 94% | 2548 (11%) |
| No – looking for a job | 236 | 1% |  |
| No – retired or is a carer | 994 | 5% |  |

|  | **2-class** | **3-class** | **4-class** | **5-class** | **6-class** |
| --- | --- | --- | --- | --- | --- |
| **AIC** | 408121.365 | 404886.908 | 402831.547 | **401506.014** | 400595.410 |
| **BIC** | 408369.847 | 405191.500 | 403192.247 | **401922.823** | 401068.329 |
| **Proportions** | 56% | 47% | 41% | **41%** | 39% |
|  | 44% | 34% | 34% | **35%** | 34% |
|  |  | 19% | 18% | **13%** | 12% |
|  |  |  | 7% | **7%** | 9% |
|  |  |  |  | **3%** | 3% |
|  |  |  |  |  | 1% |
| **Entropy** | 0.58 | 0.68 | 0.73 | **0.74** | 0.76 |
| **Classification accuracy** | 90% | 89% | 87% | **87%** | 87% |
|  | 85% | 79% | 79% | **81%** | 82% |
|  |  | 90% | 90% | **77%** | 76% |
|  |  |  | 88% | **74%** | 75% |
|  |  |  |  | **87%** | 88% |
|  |  |  |  |  | 92% |
| **BLRT** | *p* <0.05 | *p* <0.05 | *p* <0.05 | ***p* <0.05** | *-* |

*Table S3: Latent Class Analysis Results*

|  | **Non-working families**  (3%) | **Affluent-families in deprived-schools'** (13%) | **Lower Affluence families**  (35%) | **Higher Affluence families**  (41%) | **Deprived families**  (7%) | **Sample average**  (100%) |
| --- | --- | --- | --- | --- | --- | --- |
| **FSM** | | | | | | |
| Mean | 16% | 30% | 15% | 12% | 34% | 17% |
| 95% CI | 15 – 16% | 30 – 31% | 15 - 15% | 12 - 12% | 34 - 35% | 17 - 17% |
| Std. dv | 5.80 | 4.63 | 5.19 | 4.81 | 6.47 | 9.07 |
| **Family Affluence Scale** | | | | | |  |
| *Do you have access to a car?* | | | | | |  |
| No | 6% | 3% | 8% | 1% | 15% | 5% |
| Yes, one | 45% | 27% | 49% | 10% | 58% | 30% |
| Yes, two or more | 49% | 71% | 42% | 89% | 27% | 65% |
| *Do you have your own bedroom?* | | | | | |  |
| No | 20% | 11% | 22% | 5% | 34% | 14% |
| Yes | 80% | 89% | 78% | 95% | 67% | 85% |
| *How many computers do you have at home?* | | | | | |  |
| None | 2% | 2% | 3% | 1% | 4% | 2% |
| One | 11% | 7% | 12% | 4% | 16% | 8% |
| Two | 20% | 15% | 21% | 9% | 25% | 16% |
| More than Two | 67% | 77% | 65% | 86% | 56% | 75% |
| *Do you have a dishwasher at home?* | | | | | |  |
| No | 55% | 32% | 61% | 12% | 77% | 38% |
| Yes | 45% | 68% | 39% | 88% | 24% | 62% |
| *How many bathrooms do you have at home?* | | | | | |  |
| None | <1% | <1% | <1% | <1% | 1% | <1% |
| One | 65% | 44% | 70% | 21% | 81% | 47% |
| Two | 28% | 41% | 25% | 45% | 15% | 34% |
| More than two | 7% | 15% | 6% | 35% | 3% | 19% |
| *How many holidays have you been on this year?* | | | | | |  |
| Not at all | 18% | 11% | 19% | 6% | 26% | 13% |
| Once | 31% | 25% | 32% | 17% | 35% | 24% |
| Twice | 24% | 25% | 23% | 22% | 20% | 23% |
| More than twice | 28% | 40% | 26% | 55% | 19% | 40% |
| **Mother's employed** | | | | | |  |
| Yes | <1% | 91% | 86% | 95% | 56% | 85% |
| No – looking for a job | <1% | 2% | 5% | 1% | 8% | 3% |
| No – retired or is a carer | 100% | 7% | 10% | 5% | 36% | 12% |
| **Father's employed** | | | | | |  |
| Yes | <1% | 99% | 97% | 99% | 78% | 94% |
| No – looking for a job | 1% | 1% | 2% | <1% | 5% | 1% |
| No – retired or is a carer | 99% | 0% | 1% | 1% | 18% | 5% |

*Table S4: Latent class analysis - socioeconomic status measures mean and proportions*

|  | **Mental Wellbeing** | | | **Internalising symptoms** | | | **Ever Smoked** | **Ever used Alcohol** | **Ever used Cannabis** | **Regularly Smoked** | **Ever got Drunk?** | **Past-month Cannabis** |
| --- | --- | --- | --- | --- | --- | --- | --- | --- | --- | --- | --- | --- |
|  | *Mean* | *St. Dev* | *CI 95%* | *Mean* | *St. Dev* | *CI 95%* | *% ‘Yes’* | *% ‘Yes’* | *% ‘Yes’* | *% ‘Yes’* | *% ‘Yes’* | *% 'Yes'* |
| **Non-working families** | **20.86** | **4.44** | **20.60 – 21.25%** | **7.12** | **4.82** | **6.67 – 7.37%** | **20%** | **47%** | **12%** | **7%** | **13%** | **8%** |
| **Affluent-families in deprived-schools families** | 21.81 | 4.45 | 21.74 – 22.06% | 5.33 | 4.44 | 5.44 – 5.77% | 14% | 44% | 8% | 2% | 10% | 4% |
| **Lower affluence**   **families** | 21.36 | 4.48 | 21.47 – 21.67% | 6.26 | 4.52 | 5.83 – 6.03% | 14% | 40% | 9% | 4% | 9% | 5% |
| **Higher affluence**   **families** | 22.61 | 4.50 | 22.30 – 22.48% | 4.95 | 4.31 | 5.14 – 5.32% | 10% | 43% | 7% | 2% | 9% | 4% |
| **Deprived families** | 21.14 | 4.70 | 20.78 – 21.27% | 6.69 | 4.74 | 6.11 – 6.60% | 16% | 40% | 11% | 5% | 10% | 7% |

*Table S5: Distal outcomes of latent classes; values in bold show the poorest outcome*

*Table S6: Equality tests of means and Wald tests*

|  | **Mental wellbeing** | **Internalising symptoms** | **Ever Smoked** | **Ever used Alcohol** | **Ever used Cannabis** | **Regularly Smoked** | **Ever got Drunk** | **Past-month Cannabis** |
| --- | --- | --- | --- | --- | --- | --- | --- | --- |
| **Overall test** | ***χ^2^* = 238.96,**  ***p*<0.05** | ***χ^2^* = 253.79,**  ***p*<0.05** | ***χ^2^* = 253.79,**  ***p*<0.05** | ***χ^2^* = 20.28,**  ***p*<0.05** | ***χ^2^* = 35.90,**  ***p*<0.05** | ***χ^2^* = 50.99,**  ***p*<0.05** | *χ^2^* = 8.63,  *p*=0.07 | ***χ^2^* = 30.50,**  ***p*<0.05** |
| **Lower affluence vs. Higher Affluence** | ***χ^2^* = 161.53,**  ***p*<0.05** | ***χ^2^* = 141.39,**  ***p*<0.05** | ***χ^2^* = 37.70,**  ***p*<0.05** | ***χ^2^* = 9.81,**  ***p*<0.05** | ***χ^2^* = 9.41,**  ***p*<0.05** | ***χ^2^* = 23.01,**  ***p*<0.05** | *χ^2^* = 0.25,  *p*=0.62 | ***χ^2^* = 5.99,**  ***p*<0.05** |
| **Lower Affluence vs. Deprived** | *χ^2^* = 1.23,  *p*=0.27 | ***χ^2^* = 5.05,**  ***p*<0.05** | *χ^2^* = 2.53,  *p*=0.11 | *χ^2^* = 0.08,  *p*=0.78 | ***χ^2^* = 5.05,**  ***p*<0.05** | *χ^2^* = 1.57,  *p*=0.21 | *χ^2^* = 0.67,  *p*=0.41 | ***χ^2^* = 4.92,**  ***p*<0.05** |
| **Lower Affluence vs. Non-working** | ***χ^2^* = 6.74,**  ***p*<0.05** | ***χ^2^* = 15.42,**  ***p*<0.05** | ***χ^2^* = 14.63,**  ***p*<0.05** | ***χ^2^* = 11.16,**  ***p*<0.05** | ***χ^2^* = 7.17,**  ***p*<0.05** | ***χ^2^* = 9.21,**  ***p*<0.05** | ***χ^2^* = 7.27,**  ***p*<0.05** | ***χ^2^* = 9.98,**  ***p*<0.05** |
| **Lower Affluence vs. Affluent-families in deprived-schools** | ***χ^2^* = 12.95,**  ***p*<0.05** | ***χ^2^* = 38.24,**  ***p*<0.05** | *χ^2^* = 0.05,  *p*=0.95 | ***χ^2^* = 7.99,**  ***p*<0.05** | *χ^2^* = 0.01,  *p*=0.92 | ***χ^2^* = 9.29,**  ***p*<0.05** | *χ^2^* = 1.37,  *p*=0.24 | *χ^2^* = 1.61,  *p*=0.21 |
| **Higher Affluence vs. Deprived** | ***χ^2^* = 44.83,**  ***p*<0.05** | ***χ^2^* = 91.18,**  ***p*<0.05** | ***χ^2^* =** **24.76,**  ***p*<0.05** | ***χ^2^* = 4.64,**  ***p*<0.05** | ***χ^2^* = 15.31,**  ***p*<0.05** | ***χ^2^* = 14.24,**  ***p*<0.05** | *χ^2^* = 0.32,  *p*=0.58 | ***χ^2^* = 12.37,**  ***p*<0.05** |
| **Higher Affluence vs. Non-working** | ***χ^2^* = 86.56,**  ***p*<0.05** | ***χ^2^* = 104.22,**  ***p*<0.05** | ***χ^2^* = 41.06,**  ***p*<0.05** | *χ^2^* = 3.48,  *p*=0.06 | ***χ^2^* = 15.80,**  ***p*<0.05** | ***χ^2^* = 22.12,**  ***p*<0.05** | ***χ^2^* = 6.40,**  ***p*<0.05** | ***χ^2^* = 16.70,**  ***p*<0.05** |
| **Higher Affluence vs. Affluent-families in deprived-schools** | ***χ^2^* = 44.83,**  ***p*<0.05** | ***χ^2^* = 7.20,**  ***p*<0.05** | ***χ^2^* = 21.33,**  ***p*<0.05** | *χ^2^* = 0.34,  *p*=0.56 | ***χ^2^* = 4.72,**  ***p*<0.05** | *χ^2^* = 0.26,  *p*=0.61 | *χ^2^* = 0.73,  *p*=0.39 | *χ^2^* = 0.26,  *p*=0.61 |
| **Deprived vs. Non-working** | *χ^2^* = 1.13,  *p*=0.29 | *χ^2^* = 2.55,  *p*=0.11 | ***χ^2^* =** **4.61,**  ***p*<0.05** | ***χ^2^* = 8.47,**  ***p*<0.05** | *χ^2^* = 0.51,  *p*=0.48 | *χ^2^* = 3.51,  *p*=0.06 | *χ^2^* = 3.02,  *p*=0.08 | *χ^2^* = 1.60,  *p*=0.21 |
| **Deprived vs. Affluent-families in deprived-schools** | ***χ^2^* = 8.18,**  ***p*<0.05** | ***χ^2^* = 32.34,**  ***p*<0.05** | *χ^2^* = 1.60,  *p*=0.21 | ***χ^2^* = 4.10,**  ***p*<0.05** | *χ^2^* = 3.66,  *p*=0.06 | ***χ^2^* = 7.72,**  ***p*<0.05** | *χ^2^* = 0.01,  *p*=0.93 | ***χ^2^* = 6.55,**  ***p*<0.05** |
| **Non-working vs. Affluent-families in deprived-schools** | ***χ^2^* = 21.33,**  ***p*<0.05** | ***χ^2^* = 56.97,**  ***p*<0.05** | ***χ^2^* = 12.66,**  ***p*<0.05** | *χ^2^* = 1.81,  *p*=0.18 | ***χ^2^* = 6.61,**  ***p*<0.05** | ***χ^2^* = 18.34,**  ***p*<0.05** | *χ^2^* = 3.45,  *p*=0.06 | ***χ^2^* = 13.15,**  ***p*<0.05** |

**Mplus code for performing latent class analysis with a latent variable**

TITLE: LCA 5-CLASS FINAL

DATA: FILE IS “S:/LCA_data.dat”;

VARIABLE:

NAMES ARE

lang schtype ID1 ID2 id2c ID4 month YEAR sex grade ethnic fascar fasbed

fascomp fasdish fasbath fasholi drunk30 fstcig fstcan fstalc truant

excluded employfa empntfa employmo empntmo wemwbs2 intsymp wksmoke

canuser bexclude btruant FSM ethnic2 moemploy faemploy smoke_yn alc_yn

can_yn regsmoke drunk_yn regcan;

MISSING ARE ALL (-99);

IDVARIABLE IS ID4;

CATEGORICAL ARE

fascar fasbed fascomp fasdish fasbath fasholi moemploy faemploy;

USEVARIABLES ARE

fascar fasbed fascomp fasdish fasbath fasholi moemploy faemploy FSM;

CLASSES = c(5); !Always start with 2 classes

ANALYSIS:

PROCESSORS = 4 1;

ESTIMATOR = MLF;

!Changed due to saddle points in estimation suggest to use MLF estimator

!NOTE start with ML/MLR not MLF when using this syntax – Mplus will tell you to use MLF if necessary

TYPE = MIXTURE;

STARTS 1000 100;

STITERATIONS = 50;

OPTSEED = 659773;

!Added in for best LL -200701.014 - repeated 6 times, see 1st run. Bengt advises 5+ - see Asparouhov and Muthén (2012) on how to do this

LRTSTARTS = 0 0 2000 400;

!Added in for TECH14

MODEL:

%OVERALL%

FAS BY fascar fasbed fascomp fasdish fasbath fasholi;

%C#1%

[FAS*1];

!This is the latent variable, you can leave this blank otherwise

PLOT:

TYPE = PLOT3;

SERIES = fascar(0) fasbed(1) fascomp(2) fasdish(3) fasbath(4) fasholi(5)

moemploy(6) faemploy(7) | FSM(0);

!This shows a graph

OUTPUT: TECH14

!This will give you the BLRT likelihood ratio tests

!Use TECH11 also if you are using a ML or MLR estimator

SAVEDATA:

SAVE=CPROB;

FILE IS SES_LCA_5C.txt;

!This will save the predicted probabilities of classes and best class fit if you want them

**Mplus code - binary outcomes: ever used alcohol as an example**

TITLE: LCA DCATEGORICAL FOR BINARY OUTCOMES

DATA: FILE IS “S:/LCA_data.dat”;

VARIABLE:

NAMES ARE

lang schtype ID1 ID2 id2c ID4 month YEAR sex grade ethnic fascar fasbed

fascomp fasdish fasbath fasholi drunk30 fstcig fstcan fstalc truant

excluded employfa empntfa employmo empntmo wemwbs2 intsymp wksmoke

canuser bexclude btruant FSM ethnic2 moemploy faemploy smoke_yn alc_yn

can_yn regsmoke drunk_yn regcan;

MISSING ARE ALL (-99);

IDVARIABLE IS ID4;

CATEGORICAL ARE

fascar fasbed fascomp fasdish fasbath fasholi moemploy faemploy;

AUXILIARY = alc_yn (DCATEGORICAL);

!Added distal outcome - equal variances

USEVARIABLES ARE

fascar fasbed fascomp fasdish fasbath fasholi moemploy faemploy FSM;

CLASSES = c(5); !Always start with 2 classes

ANALYSIS:

PROCESSORS = 4 1;

ESTIMATOR = MLF;

!changed due to saddle points in estimation – use ML/MLR if you are starting your LCA model – only use MLF if Mplus says too

TYPE = MIXTURE;

STARTS 100 30;

MODEL:

%OVERALL%

FAS BY fascar fasbed fascomp fasdish fasbath fasholi;

%C#1%

[FAS*1];

!This is the latent variable, you can leave this blank otherwise

OUTPUT: ;

**Mplus code - continuous distal outcomes: mental wellbeing as an example**

TITLE: LCA DU3STEP FOR CONTINUOUS OUTCOMES

DATA: FILE IS “S:/LCA_data.dat”;

VARIABLE:

NAMES ARE

lang schtype ID1 ID2 id2c ID4 month YEAR sex grade ethnic fascar fasbed

fascomp fasdish fasbath fasholi drunk30 fstcig fstcan fstalc truant

excluded employfa empntfa employmo empntmo wemwbs2 intsymp wksmoke

canuser bexclude btruant FSM ethnic2 moemploy faemploy smoke_yn alc_yn

can_yn regsmoke drunk_yn regcan;

MISSING ARE ALL (-99);

IDVARIABLE IS ID4;

CATEGORICAL ARE

fascar fasbed fascomp fasdish fasbath fasholi moemploy faemploy;

AUXILIARY = wemwbs2(DU3STEP); !Added distal outcome

USEVARIABLES ARE

fascar fasbed fascomp fasdish fasbath fasholi moemploy faemploy FSM;

CLASSES = c(5); !Always start with 2 classes

ANALYSIS:

PROCESSORS = 4 1;

ESTIMATOR = MLF;

!changed due to saddle points in estimation – use ML/MLR if you are starting your LCA model – only use MLF if Mplus says too

TYPE = MIXTURE;

STARTS 100 30;

MODEL:

%OVERALL%

FAS BY fascar fasbed fascomp fasdish fasbath fasholi;

%C#1%

[FAS*1];

!This is the latent variable, you can leave this blank otherwise

OUTPUT: cinterval;

!This will give you confidence intervals if you want them

**Mplus code - Covariates to predict classes**

TITLE: LCA – R3STEP FOR OR’S OF CLASSES *ON* COVARIATES

DATA: FILE IS “S:/LCA_data.dat”;

VARIABLE:

NAMES ARE

lang schtype ID1 ID2 id2c ID4 month YEAR sex grade ethnic fascar fasbed

fascomp fasdish fasbath fasholi drunk30 fstcig fstcan fstalc truant

excluded employfa empntfa employmo empntmo wemwbs2 intsymp wksmoke

canuser bexclude btruant FSM ethnic2 moemploy faemploy smoke_yn alc_yn

can_yn regsmoke drunk_yn regcan ethnic7 gender;

MISSING ARE ALL (-99);

AUXILIARY = grade (R3STEP) ethnic7 (R3STEP) gender (R3STEP);

IDVARIABLE IS ID4;

CATEGORICAL ARE

fascar fasbed fascomp fasdish fasbath fasholi moemploy faemploy;

USEVARIABLES ARE

fascar fasbed fascomp fasdish fasbath fasholi moemploy faemploy FSM;

CLASSES = c(5); !Always start with 2 classes

ANALYSIS:

PROCESSORS = 4 1;

ESTIMATOR = MLF;

!changed due to saddle points in estimation suggest to use MLF estimator - use ML/MLR if you are starting your LCA model – only use MLF if Mplus says too

TYPE = MIXTURE;

STARTS 1000 100;

STITERATIONS = 50;

MODEL:

%OVERALL%

FAS BY fascar fasbed fascomp fasdish fasbath fasholi;

%C#1%

[FAS*1];

OUTPUT: ;
